# Association of systemic inflammation indices with micronutrient status in adult patients with celiac disease

**DOI:** 10.3389/fmed.2026.1898592

**Published:** 2026-10-19

**Authors:** Selçuk Candan, Okan Katı, Tuğçe Eşkazan, Oğuz Kağan Bakkaloğlu, İbrahim Hatemi, Ahmet Merih Dobrucalı, Billur Canbakan

**Affiliations:** Department of Gastroenterology, Cerrahpaşa Faculty of Medicine, Istanbul University-Cerrahpaşa, Istanbul, Türkiye

**Keywords:** celiac disease, iron deficiency, micronutrient status, systemic immune-inflammation index, vitamin B12 deficiency

## Abstract

**Background:**

Celiac disease is a chronic immune-mediated enteropathy associated with micronutrient deficiencies and persistent inflammation. Systemic inflammation indices derived from complete blood count parameters may reflect systemic inflammatory activity, but their relationship with micronutrient status in adult celiac disease remains unclear. We evaluated the association between systemic inflammation indices and micronutrient status in adults with celiac disease.

**Materials and methods:**

This retrospective cross-sectional study included 120 adults with celiac disease followed at a tertiary center between January 2020 and October 2023. Systemic immune-inflammation index (SII), neutrophil-to-lymphocyte ratio (NLR), and platelet-to-lymphocyte ratio (PLR) were evaluated according to ferritin, transferrin saturation, vitamin B12, folate, and vitamin D status. Multiple comparisons were adjusted using the Holm method, and Firth penalized logistic regression was used for multivariable analyses of ferritin and vitamin B12 deficiency.

**Results:**

Patients with ferritin deficiency had higher SII, NLR, and PLR values, and all associations remained significant after Holm correction. Higher SII and NLR were observed in patients with low transferrin saturation and vitamin B12 deficiency in unadjusted analyses, but these associations did not remain significant after Holm correction. In Firth penalized logistic regression, higher SII was associated with ferritin deficiency (adjusted OR per doubling of SII, 2.12; 95% CI, 1.31–3.58; *p* = 0.002). The association with vitamin B12 deficiency was considered exploratory because of the limited number of events. No associations were detected for folate or vitamin D deficiency.

**Conclusion:**

Higher systemic inflammation indices, particularly SII, were associated with reduced iron stores in adults with celiac disease, with the association between SII and ferritin deficiency persisting after multivariable adjustment. Associations with transferrin saturation and vitamin B12 did not remain significant after correction for multiple comparisons and should be interpreted cautiously. These findings support further investigation of the relationship between systemic inflammation and micronutrient status in larger prospective cohorts.

## Introduction

Celiac disease is a chronic, immune-mediated enteropathy that develops in genetically susceptible individuals following exposure to dietary gluten. It is characterized by varying degrees of small-intestinal mucosal injury, including villous atrophy, crypt hyperplasia and increased intraepithelial lymphocytosis (1). These structural alterations may lead to impaired absorption of several micronutrients, giving rise to a broad spectrum of gastrointestinal and extraintestinal manifestations (2). In adults, the disease often presents with nonspecific symptoms, biochemical abnormalities or isolated anemia, and is increasingly recognized beyond childhood (3).

Systemic and mucosal inflammation plays a central role in the pathogenesis and clinical course of celiac disease (4). In recent years, inflammation-based indices derived from peripheral blood counts have attracted growing interest as measures of systemic immune-inflammatory activity. Among these, the systemic immune-inflammation index (SII)**,** which integrates neutrophil, lymphocyte and platelet counts into a single composite parameter, has emerged as a relatively new and comprehensive marker that may reflect the balance between immune response, inflammation and platelet activation (5, 6). Traditional indices such as the neutrophil-to-lymphocyte ratio (NLR) and platelet-to-lymphocyte ratio (PLR) have been investigated in various chronic inflammatory and autoimmune disorders; however, data on SII in celiac disease, particularly in relation to nutritional parameters, remain limited (7, 8). Compared with NLR and PLR, SII incorporates three peripheral blood cell populations (neutrophils, lymphocytes, and platelets) and therefore captures a different composite representation of systemic immune-inflammatory status. For this reason, SII was selected as the primary inflammatory index in the present study, whereas NLR and PLR were included as established comparator indices.

Micronutrient deficiencies are common in celiac disease and may persist despite diagnosis and dietary treatment (9). In the present study, ferritin and transferrin saturation were selected to assess iron status because they provide complementary information regarding iron stores and functional iron availability, particularly in the presence of chronic inflammation (9, 10). Vitamin B12 and folate were included because deficiencies may result from impaired intestinal absorption and are associated with hematological and neurological complications, whereas vitamin D was evaluated because of its established role in bone metabolism and immune regulation. Collectively, these micronutrient abnormalities may contribute to anemia, fatigue, reduced quality of life, and adverse long-term clinical outcomes in patients with celiac disease (11).

The interaction between systemic inflammation and micronutrient status in celiac disease is complex and not yet fully clarified. Chronic inflammation may disturb iron homeostasis through hepcidin-mediated pathways, resulting in functional iron deficiency, while persistent micronutrient deficiencies may, in turn, sustain low-grade inflammatory activity (12). In this context, SII and other inflammation-based indices may provide additional measures for investigating the relationship between systemic inflammatory activity and micronutrient disturbances (13)**.** Nevertheless, studies evaluating SII in conjunction with ferritin, transferrin saturation and vitamin levels in adult patients with celiac disease are scarce (14).

Iron deficiency and vitamin B12 deficiency may influence systemic immune responses through different biological mechanisms. Chronic intestinal inflammation promotes cytokine-mediated alterations in iron metabolism, particularly via hepcidin-induced iron sequestration, whereas vitamin B12 deficiency may reflect more extensive mucosal damage, prolonged malabsorption, and sustained immune activation. In contrast, vitamin D and folate status are influenced by multiple non-inflammatory factors, including dietary intake, supplementation, sunlight exposure, and individual nutritional habits. Therefore, the relationship between systemic inflammation indices and individual micronutrients is likely to differ according to their underlying pathophysiological mechanisms. This biological heterogeneity provides a rationale for evaluating each micronutrient separately rather than assuming a uniform relationship with systemic inflammatory indices.

The present study aimed to comprehensively investigate the relationship between the systemic immune-inflammation index (SII) and micronutrient status in adult patients with celiac disease. Emphasis was placed on parameters related to iron metabolism (ferritin and transferrin saturation) and vitamin B12 levels**.** NLR and PLR were additionally evaluated as supportive indices for comparison, while vitamin D and folate levels were considered secondary variables within the scope of the study (13, 15)**.**

## Materials and methods

### Study design and population

This retrospective cross-sectional study included adult patients with celiac disease who were followed at the gastroenterology outpatient clinic of a tertiary care center between January 2020 and October 2023. The study cohort was retrospectively assembled from patients under regular outpatient follow-up who met the predefined eligibility criteria and had sufficient clinical and laboratory data available for analysis. Patients meeting the predefined exclusion criteria described below were not entered into the study dataset during retrospective data collection. Consequently, the total number of excluded patients and the number excluded for each individual reason could not be reliably reconstructed. Patients aged 18 years or older with biopsy- and/or serology-proven celiac disease were eligible. The diagnosis was based on compatible clinical findings together with positive celiac-specific serology and histopathological features consistent with celiac disease in accordance with current guideline recommendations (2).

Patients with acute infection at the time of sampling, chronic inflammatory or autoimmune diseases (including inflammatory bowel disease, rheumatoid arthritis, systemic lupus erythematosus, and systemic vasculitis), malignant disease, hematological disorders unrelated to celiac disease, chronic liver or renal failure, or missing key laboratory data were excluded. Information regarding iron, vitamin B12, and folate supplementation was obtained from the available medical records. Patients receiving active iron, vitamin B12, or folate replacement therapy at the time of laboratory assessment were excluded to minimize potential confounding.

### Data collection

Demographic data (age, sex), body mass index (BMI), family history of celiac disease, smoking status, gluten-free diet adherence, and relevant clinical information were obtained from medical records. Family history of celiac disease was defined as a self-reported history of biopsy- or physician-diagnosed celiac disease in a first-degree relative (parent, sibling, or offspring), as documented in the medical records. Laboratory parameters measured at routine follow-up visits included complete blood count, serum ferritin, serum iron, total iron-binding capacity (TIBC), vitamin B12, folate, and 25-hydroxyvitamin D levels. Complete blood count parameters were measured using a Sysmex XN-1000 automated hematology analyzer (Sysmex Corporation, Kobe, Japan) using light scattering, electrical impedance, and photometric methods. Serum ferritin, vitamin B12, folate, and 25-hydroxyvitamin D concentrations were measured using electrochemiluminescence immunoassays (ECLIA) on the Cobas 8000 e801 platform (Roche Diagnostics, Mannheim, Germany). Transferrin saturation was calculated from serum iron and total iron-binding capacity values as follows: transferrin saturation (%) = (serum iron/TIBC) × 100. All laboratory measurements were performed in the hospital's central laboratory according to the manufacturers’ instructions and routine internal quality-control procedures.

Adherence to a gluten-free diet was assessed by the treating physician during routine outpatient follow-up and documented in the medical records as a binary variable (yes/no). The assessment was based on routine clinical evaluation rather than a standardized validated adherence instrument or serological marker. Therefore, GFD adherence was interpreted cautiously in the present study.

### Inflammation-based indices

Systemic inflammation indices were calculated from complete blood count parameters using the following formulas:

Systemic immune-inflammation index (SII) (5):

SII = platelet count (×10⁹/L) × neutrophil count (×10⁹/L)/lymphocyte count (×10⁹/L)

Neutrophil-to-lymphocyte ratio (NLR) (7):

NLR = neutrophil count (×10⁹/L)/lymphocyte count (×10⁹/L)

Platelet-to-lymphocyte ratio (PLR) (16):

PLR = platelet count (×10⁹/L)/lymphocyte count (×10⁹/L)

SII was regarded as the primary inflammation-based index, while NLR and PLR were evaluated as comparator indices. This approach was chosen because SII integrates neutrophil, lymphocyte, and platelet counts into a single composite index, whereas NLR and PLR are based on two-cell ratios.

### Micronutrient status

Micronutrient deficiencies were defined using the reference ranges routinely applied by the hospital's central laboratory: serum ferritin <15 ng/mL, transferrin saturation <20%, vitamin B12 < 200 pg/mL, folate <4 ng/mL, and 25-hydroxyvitamin D < 20 ng/mL. These cut-off values are consistent with internationally accepted guideline recommendations (17–19).

Vitamin D and folate were considered secondary variables within the scope of the study.

### Definition of anemia

Anemia was defined in accordance with World Health Organization criteria as hemoglobin <13 g/dL in men and <12 g/dL in women. Anemia was evaluated as a secondary clinical finding rather than a primary study outcome (20).

### Statistical analysis

Statistical analyses were performed using IBM SPSS Statistics, version 26.0 (IBM Corp., Armonk, NY, USA). The distribution of continuous variables was assessed using visual inspection and the Shapiro–Wilk test. Continuous variables were expressed as mean ± standard deviation or median (interquartile range), as appropriate. Group comparisons were performed using the independent samples t-test or Mann–Whitney U test according to data distribution. For non-parametric group comparisons, effect sizes were expressed as Cliff's delta (*δ*) with 95% confidence intervals obtained using bootstrap resampling. Associations between systemic inflammation indices (SII, NLR, and PLR) and micronutrient deficiencies were evaluated using univariate analyses. Correlations between serum ferritin levels and systemic inflammation indices were assessed using Spearman's rank correlation coefficient.

To account for the limited number of deficiency events and reduce small-sample bias, Firth penalized logistic regression was used to evaluate the associations of SII with ferritin deficiency and vitamin B12 deficiency. Because SII showed a right-skewed distribution, it was log₂-transformed before inclusion in the regression models; accordingly, odds ratios represent the change in odds associated with a doubling of SII. Models were adjusted for age, sex, body mass index (BMI), gluten-free diet adherence, and smoking status. Adjusted odds ratios (ORs) with 95% confidence intervals (CIs) were reported. Given the limited number of vitamin B12 deficiency events, the corresponding analysis was considered exploratory.

As a sensitivity analysis addressing the potential information loss associated with dichotomization, serum ferritin and vitamin B12 concentrations were additionally analyzed as continuous outcomes using multivariable linear regression. Because of their right-skewed distributions, ferritin and vitamin B12 concentrations were natural-log transformed. The same covariates were included in these models, with log₂-transformed SII as the primary explanatory variable. Regression coefficients (*β*) with 95% CIs were reported. Model diagnostics included assessment of multicollinearity using variance inflation factors (VIFs), heteroscedasticity, and influential observations. Sensitivity analyses using heteroscedasticity-robust standard errors and exclusion of influential observations were performed where appropriate.

The primary hypothesis was that higher SII would be associated with poorer micronutrient status, particularly lower iron stores as reflected by ferritin, in adults with celiac disease. Associations with transferrin saturation and vitamin B12 were also evaluated as primary micronutrient outcomes, whereas NLR and PLR were examined as comparator inflammatory indices. Folate and vitamin D were considered secondary micronutrient outcomes. To account for multiple testing across the 15 univariate comparisons of SII, NLR, and PLR with the five micronutrient deficiency categories, *p* values were adjusted using the Holm method. Both unadjusted and Holm-adjusted *p* values are reported, with statistical significance for these comparisons determined on the basis of the Holm-adjusted *p* values. Analyses were performed using available-case data for each variable; participants with missing data for a given laboratory parameter or inflammatory index were excluded only from the corresponding analysis, and no imputation of missing values was performed. Accordingly, sample sizes varied across analyses and are reported for each comparison. A two-sided *p* value <0.05 was considered statistically significant.

### Ethics approval

The study was reviewed and approved by the Medical Research Ethics Committee of Istanbul University-Cerrahpaşa (approval no. 1620074; approval date: 14 April 2026). The study was conducted in accordance with the Declaration of Helsinki. Owing to the retrospective design of the study and the use of anonymized clinical data, the requirement for written informed consent was waived by the ethics committee.

## Results

A total of 120 adult patients with celiac disease were included in the analysis. The mean age of the study population was 35.3 ± 15.5 year**s** (median 30.5 years). Of these, 79 (65.8%) were female and 41 (34.2%) were male. The mean body mass index (BMI) was 23.4 ± 2.8 kg/m^2^ (median 23.1 kg/m^2^). The median disease duration was 9 years [interquartile range (IQR), 6–13 years] calculated from the age at diagnosis to the time of study inclusion. Adherence to a gluten-free diet was documented in 108 (90.0%) patients, whereas 12 (10.0%) patients were non-adherent.

A family history of celiac disease was present in 17 (14.2%) patients. Regarding smoking status, 82 (68.3%) were non-smokers, 17 (14.2%) were current smokers and 21 (17.5%**)** were former smokers. Diarrhea was reported by 43 (35.8%) patients, while abdominal pain was recorded in 11 (9.2%) patients. The baseline demographic and clinical characteristics of the study population are summarized in Table 1.

**Table 1:** Demographic and baseline characteristics of the study population.

Characteristic	Value
Number of patients, n	120
Age, mean ± SD (years)	35.3 ± 15.5
Age, median (IQR) (years)	30.5 (24.0–47.0)
Sex, *n* (%)	
Female	79 (65.8%)
Male	41 (34.2%)
BMI, mean ± SD (kg/m^2^)	23.4 ± 2.8
BMI, median (IQR) (kg/m^2^)	23.1 (21.5–25.0)
Disease duration, median (IQR) (years)	9 (6–13)
Gluten-free diet adherence, *n* (%)	
Yes	108 (90.0%)
No	12 (10.0%)
Smoking status, *n* (%)	
Non-smoker	82 (68.3%)
Current smoker	17 (14.2%)
Former smoker	21 (17.5%)
Family history of celiac disease, *n* (%)	17 (14.2%)
Presenting symptoms, *n* (%)	
Diarrhea	43 (35.8%)
Abdominal pain	11 (9.2%)

When systemic inflammation indices were compared according to GFD adherence, NLR was higher in non-adherent than adherent patients (median [IQR], 2.27 [1.79–3.00] vs. 1.61 [1.24–2.32]; *p* = 0.042; Cliff's *δ*=−0.36, 95% CI −0.74 to 0.02). SII was also numerically higher among non-adherent patients, although the difference was not statistically significant (551.6 [373.7–817.5] vs. 404.0 [276.7–615.8]; *p* = 0.093; Cliff's *δ*=−0.30, 95% CI −0.65 to 0.06). No difference was detected for PLR (127.0 [117.1–156.4] vs. 119.3 [94.3–153.7]; *p* = 0.485; Cliff's *δ*=−0.12, 95% CI −0.46 to 0.21). Micronutrient deficiency rates did not differ significantly according to GFD adherence (all *p* > 0.05).

### Hematological and micronutrient findings

Anemia, defined according to WHO criteria, was present in 25 (20.8%) patients. Micronutrient measurements were not available for all participants. Ferritin measurements were available in 117 patients, of whom 38 (32.5%) had ferritin levels <15 ng/mL. Transferrin saturation could be calculated in 115 patients with available serum iron and TIBC measurements, of whom 63 (54.8%) had transferrin saturation <20%. Vitamin B12 measurements were available in 115 patients, with deficiency (<200 pg/mL) identified in 14 (12.2%). Folate measurements were available in 113 patients, with deficiency (<4 ng/mL) identified in 25 (22.1%), while 25-hydroxyvitamin D measurements were available in 97 patients, with deficiency (<20 ng/mL) identified in 66 (68.0%).

### Systemic inflammation indices

The median values of the inflammation-based indices were as follows:

NLR 1.70 (IQR 1.24–2.34), PLR 121.0 (IQR 95.0–156.5) and SII 410 (IQR 294–673).

SII values showed a relatively wide distribution and tended to be higher in patients with altered hematological or micronutrient parameters.

### Ferritin status and inflammation indices

Groupwise SII distributions according to ferritin and vitamin B12 status are presented in Figures 1 and 2, respectively. Patients with ferritin deficiency had higher SII, NLR, and PLR values than those with normal ferritin levels. These differences remained statistically significant after Holm correction for multiple comparisons (SII: Holm-adjusted *p* = 0.025, Cliff's *δ*=0.36, 95% CI 0.16–0.55; NLR: Holm-adjusted *p* = 0.049, *δ*=0.33, 95% CI 0.13–0.52; PLR: Holm-adjusted *p* = 0.007, *δ*=0.40, 95% CI 0.20–0.59) (Table 2). Spearman correlation analysis demonstrated a modest inverse correlation between serum ferritin levels and SII (*ρ*=−0.23, *p* = 0.013; *n* = 116), indicating that higher SII values tended to be associated with lower ferritin concentrations (Figure 3).

**Figure 1:**
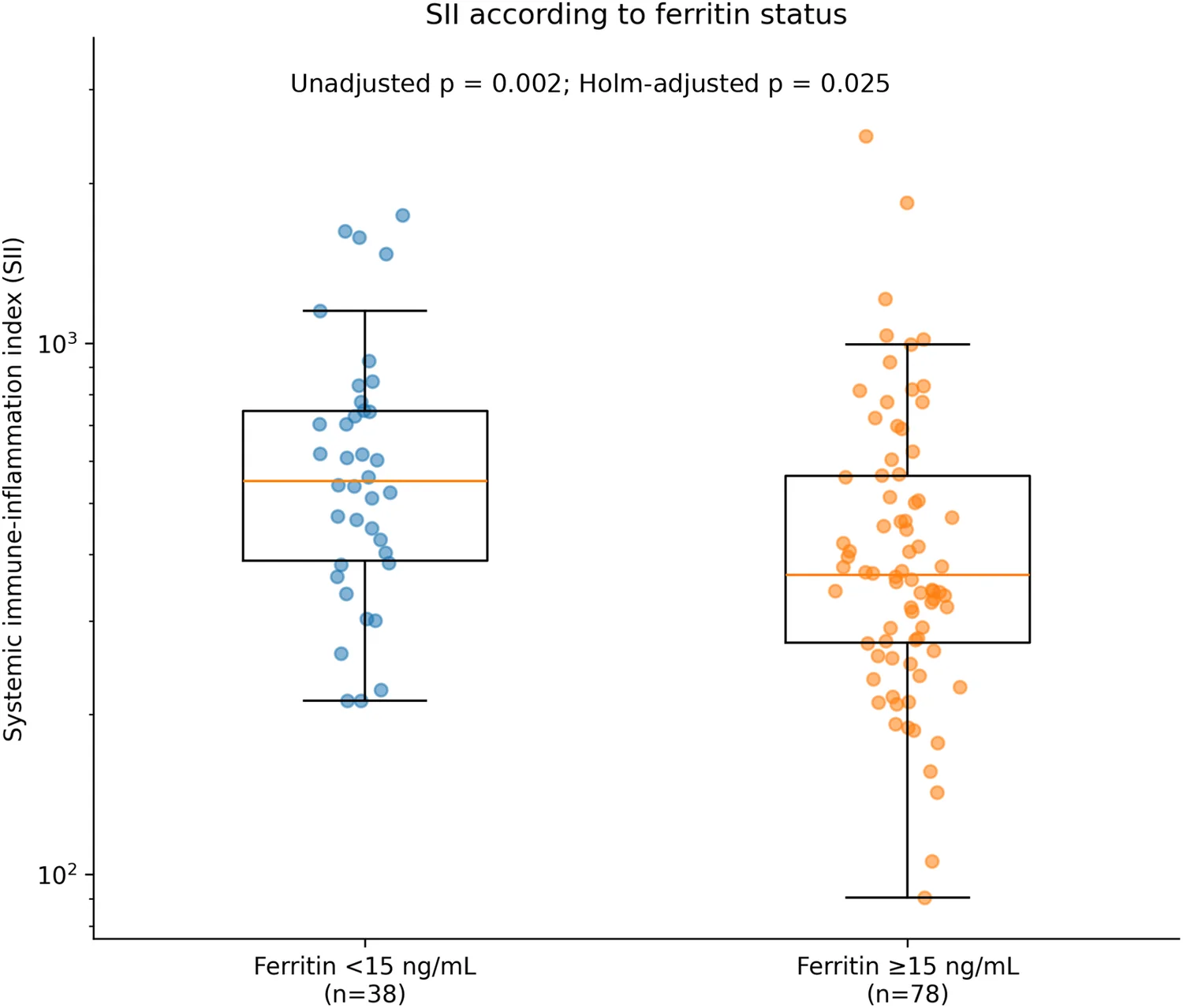
Systemic immune-inflammation index (SII) according to ferritin status. Distribution of systemic immune-inflammation index (SII) values according to ferritin status. SII values were higher in patients with ferritin deficiency (<15 ng/mL; *n* = 38) than in those with normal ferritin levels (≥15 ng/mL; *n* = 78) (unadjusted *p* = 0.002; Holm-adjusted *p* = 0.025, Mann–Whitney U test). Individual data points are shown. The *y*-axis is presented on a logarithmic scale for visualization purposes.

**Figure 2:**
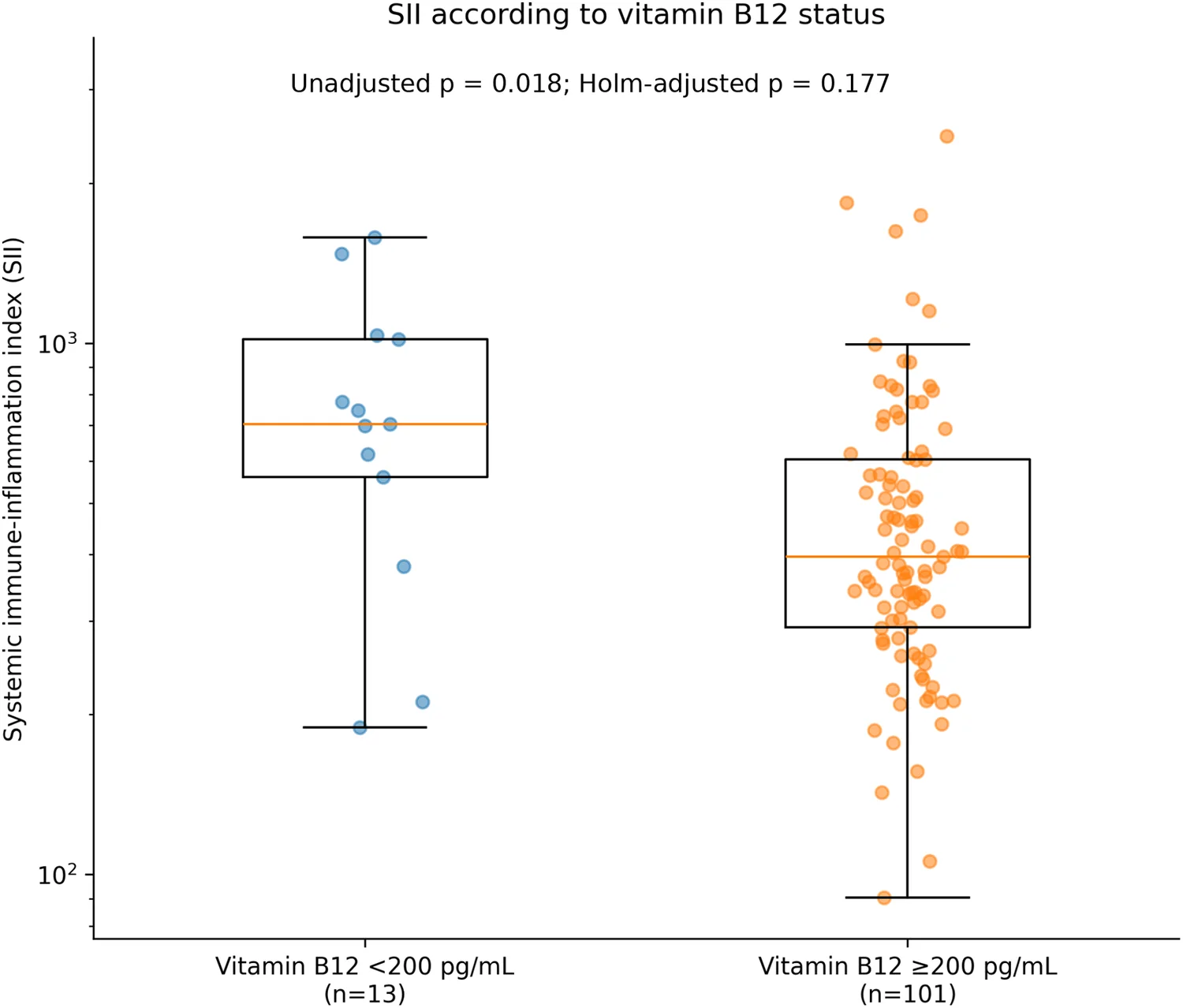
Systemic immune-inflammation index (SII) according to vitamin B12 status. Distribution of systemic immune-inflammation index (SII) values according to vitamin B12 status. SII values were higher in patients with vitamin B12 deficiency (<200 pg/mL; *n* = 13) than in those with normal vitamin B12 concentrations (≥200 pg/mL; *n* = 101) in the unadjusted analysis (*p* = 0.018); however, this difference did not remain statistically significant after correction for multiple comparisons (Holm-adjusted *p* = 0.177, Mann–Whitney U test). Individual data points are shown. The *y*-axis is presented on a logarithmic scale for visualization purposes.

**Table 2:** Comparison of systemic inflammation indices according to micronutrient status.

Micronutrient status	Index	Low/deficient group, n	Median (IQR)	Normal group, n	Median (IQR)	Unadjusted p	Holm-adjusted p	Cliff's *δ* (95% CI)
Ferritin <15 ng/mL	SII	38	550.3 (389.7–745.7)	78	365.9 (273.2–562.3)	0.002	**0** **.** **025**	0.36 (0.16–0.55)
	NLR	38	2.00 (1.44–2.77)	79	1.55 (1.17–2.21)	0.004	**0**.**049**	0.33 (0.13–0.52)
	PLR	38	145.5 (116.5–174.2)	78	116.9 (85.5–144.9)	<0.001	**0**.**007**	0.40 (0.20–0.59)
Transferrin saturation <20%	SII	63	523.1 (339.4–794.6)	52	366.8 (270.3–488.8)	0.008	0.095	0.29 (0.09–0.49)
	NLR	63	2.06 (1.40–2.70)	52	1.44 (1.21–1.97)	0.010	0.109	0.28 (0.08–0.48)
	PLR	63	126.2 (102.4–163.8)	52	117.8 (90.0–144.6)	0.095	0.757	0.18 (−0.03–0.38)
Vitamin B12 < 200 pg/mL	SII	13	704.0 (559.4–1015.3)	101	396.3 (291.8–604.8)	0.018	0.177	0.41 (0.05–0.72)
	NLR	14	2.29 (1.82–3.67)	101	1.61 (1.23–2.32)	0.041	0.368	0.34 (0.02–0.63)
	PLR	13	151.5 (107.6–177.7)	101	119.0 (94.1–151.4)	0.134	0.940	0.26 (−0.11–0.60)
Folate <4 ng/mL	SII	24	467.7 (335.7–703.6)	88	390.8 (291.6–640.9)	0.405	1.000	0.11 (−0.15–0.37)
	NLR	25	1.82 (1.25–2.31)	88	1.70 (1.24–2.38)	0.989	1.000	−0.00 (−0.27–0.26)
	PLR	24	139.9 (106.4–170.6)	88	119.3 (93.5–151.7)	0.196	1.000	0.17 (−0.10–0.43)
Vitamin D < 20 ng/mL	SII	66	459.1 (300.9–703.9)	31	396.3 (315.3–663.6)	0.484	1.000	0.09 (−0.16–0.34)
	NLR	66	1.80 (1.30–2.52)	31	1.79 (1.21–2.41)	0.562	1.000	0.07 (−0.18–0.33)
	PLR	66	123.2 (96.4–162.5)	31	120.5 (91.5–153.5)	0.802	1.000	0.03 (−0.22–0.29)

Data are presented as median (interquartile range). *P* values were obtained using the Mann–Whitney U test. To account for multiple testing across the 15 comparisons, *p* values were adjusted using the Holm method. Statistical significance was determined based on Holm-adjusted *p* values. Cliff's δ with 95% bootstrap confidence intervals was used as the effect-size measure; positive values indicate higher values of the corresponding inflammatory index in the deficient/low group. SII, systemic immune-inflammation index; NLR, neutrophil-to-lymphocyte ratio; PLR, platelet-to-lymphocyte ratio.

Bold values indicate statistically significant results after Holm adjustment (Holm-adjusted p < 0.05).

**Figure 3:**
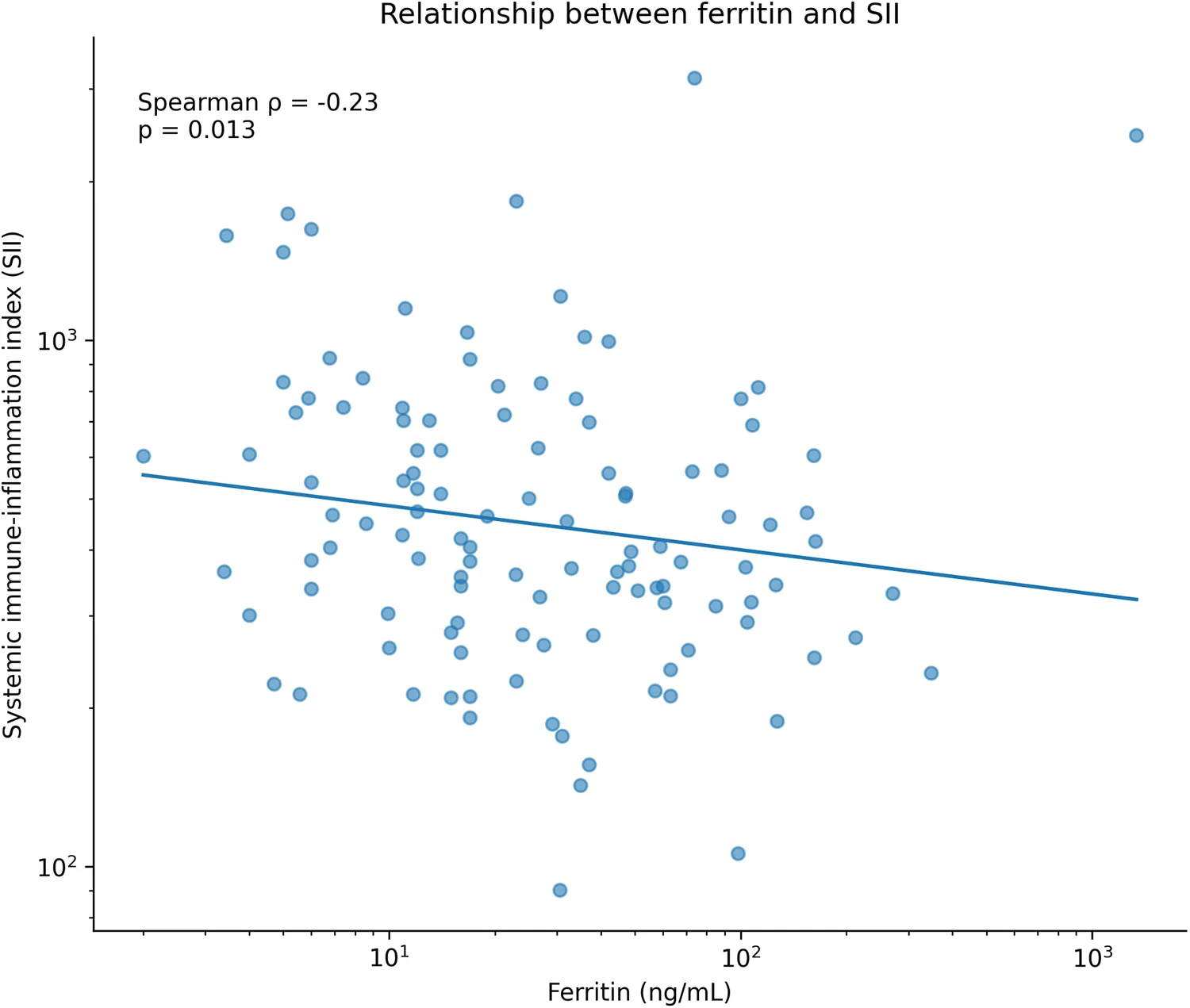
Relationship between ferritin levels and systemic immune-inflammation index (SII). Scatter plot showing the relationship between serum ferritin levels and systemic immune-inflammation index (SII). A modest inverse correlation was observed between ferritin and SII (Spearman's *ρ*=−0.23, *p* = 0.013). The solid line represents the fitted trend for visualization purposes. Both axes are presented on logarithmic scales.

### Transferrin saturation

Patients with low transferrin saturation showed higher SII and NLR values in the unadjusted analyses (SII: *p* = 0.008, Cliff's *δ*=0.29, 95% CI 0.09–0.49; NLR: *p* = 0.010, *δ*=0.28, 95% CI 0.08–0.48); however, these associations did not remain statistically significant after Holm correction (adjusted *p* = 0.095 and *p* = 0.109, respectively). No association was detected for PLR (unadjusted *p* = 0.095; Holm-adjusted *p* = 0.757; *δ*=0.18, 95% CI −0.03 to 0.38) (Table 2).

### Vitamin B12

Patients with vitamin B12 deficiency had higher SII and NLR values in the unadjusted analyses (SII: *p* = 0.018, Cliff's *δ*=0.41, 95% CI 0.05–0.72; NLR: *p* = 0.041, *δ*=0.34, 95% CI 0.02–0.63); however, neither association remained statistically significant after Holm correction (adjusted *p* = 0.177 and *p* = 0.368, respectively). No association was detected for PLR (unadjusted *p* = 0.134; Holm-adjusted *p* = 0.940; *δ*=0.26, 95% CI −0.11 to 0.60) (Table 2).

### Folate and vitamin D

No associations were detected between folate or vitamin D deficiency and SII, NLR, or PLR after correction for multiple comparisons (all Holm-adjusted *p* = 1.000). Effect sizes were small and their 95% confidence intervals included zero for all comparisons (Table 2).

### Anemia

Anemia was evaluated as a secondary clinical finding. Patients with anemia had significantly higher PLR values than non-anemic patients, while SII and NLR showed nonsignificant trends toward higher levels.

### Multivariable and sensitivity analyses

To account for the limited number of deficiency events, Firth penalized logistic regression was performed using log₂-transformed SII. After adjustment for age, sex, BMI, GFD adherence, and smoking status, higher SII remained associated with ferritin deficiency (adjusted OR per doubling of SII, 2.12; 95% CI, 1.31–3.58; *p* = 0.002). For vitamin B12 deficiency, the corresponding association was weaker but remained statistically significant (adjusted OR per doubling of SII, 1.97; 95% CI, 1.05–3.92; *p* = 0.033). Given the small number of vitamin B12-deficient patients, this finding was considered exploratory (Table 3).

**Table 3:** Firth penalized logistic regression analyses of factors associated with ferritin and vitamin B12 deficiency.

Variable	Adjusted OR (95% CI)	*p* value
Ferritin deficiency (<15 ng/mL), *n* = 116; events=38		
SII (per doubling)	**2.12** (**1.31–3.58)**	**0**.**002**
Age (per year)	0.97 (0.93–1.00)	0.065
Female sex	**3.89** (**1.30–13.01)**	**0**.**014**
BMI (kg/m^2^)	1.13 (0.94–1.38)	0.206
GFD adherence	1.57 (0.40–7.38)	0.526
Current smoker	0.44 (0.10–1.51)	0.201
Former smoker	0.86 (0.21–3.40)	0.835
Vitamin B12 deficiency (<200 pg/mL), *n* = 114; events=13		
SII (per doubling)	**1.97** (**1.05–3.92)**	**0**.**033**
Age (per year)	0.99 (0.93–1.04)	0.737
Female sex	0.38 (0.09–1.45)	0.155
BMI (kg/m^2^)	1.01 (0.78–1.32)	0.920
GFD adherence	0.65 (0.14–4.07)	0.611
Current smoker	0.49 (0.05–2.52)	0.422
Former smoker	1.65 (0.23–12.06)	0.611

BMI, body mass index; CI, confidence interval; GFD, gluten-free diet; OR, odds ratio; SII, systemic immune-inflammation index. Firth penalized logistic regression was used to reduce small-sample bias. SII was log₂-transformed before analysis; therefore, the OR for SII represents the change in odds associated with a doubling of SII. Models were adjusted for age, sex, BMI, GFD adherence, and smoking status, with male sex, GFD nonadherence, and never smoking as the reference categories. The vitamin B12 deficiency model was considered exploratory because of the limited number of deficiency events.

Bold values indicate statistically significant associations (*p* < 0.05).

In sensitivity analyses treating micronutrient concentrations as continuous outcomes, higher log₂-transformed SII was inversely associated with natural-log-transformed ferritin after multivariable adjustment (*β*=−0.274; 95% CI, −0.500 to −0.047; *p* = 0.018). An inverse association was also observed for vitamin B12 (*β*=−0.136; 95% CI, −0.223 to −0.049; *p* = 0.003). No relevant multicollinearity was detected (all VIFs <2), and there was no evidence of heteroscedasticity in either continuous-outcome model. Sensitivity analyses indicated that the vitamin B12 association was not driven by a single influential observation, whereas the continuous ferritin model showed greater sensitivity to model specification and was therefore interpreted cautiously.

## Discussion

In this study, systemic inflammation indices were evaluated in relation to micronutrient status in adults with celiac disease, with particular emphasis on SII. The most consistent associations were observed for iron status, particularly ferritin deficiency, whereas findings for transferrin saturation and vitamin B12 were less robust after correction for multiple comparisons. Overall, these findings suggest that the relationship between systemic inflammatory activity and micronutrient status may differ across individual micronutrients in treated adult celiac disease.

Previous studies in celiac disease have largely focused on NLR and PLR, reporting alterations in these indices at diagnosis and during follow-up, particularly in relation to dietary adherence (21, 22). In our cohort, NLR was higher among patients documented as non-adherent to a GFD, whereas no statistically significant differences were detected for SII or PLR. However, these findings should be interpreted cautiously because only 12 patients were classified as non-adherent and adherence was assessed from routine clinical documentation rather than a validated instrument or serological marker. In contrast, evidence regarding SII in adult celiac disease remains limited. Recent studies have suggested that SII may be associated with disease-related inflammatory activity and may have potential utility in the assessment of patients with celiac disease (23). Our findings expand upon these observations by demonstrating an association between higher SII values and micronutrient status, with the most consistent findings observed for reduced iron stores. These observations suggest a potential relationship between systemic inflammatory activity and nutritional status in celiac disease. Although similar associations between systemic inflammatory indices and micronutrient disturbances have been reported in other chronic inflammatory and autoimmune disorders, celiac disease represents a unique clinical setting because immune-mediated enteropathy, chronic malabsorption, and nutritional deficiencies coexist as integral components of the disease process (24). Consequently, alterations in SII may reflect not only systemic inflammation but also the combined effects of mucosal injury and impaired nutrient absorption, providing a disease-specific context for interpreting systemic inflammatory indices.

The multivariable analyses should be interpreted in the context of the number of deficiency events. Firth penalized logistic regression was therefore used to reduce small-sample bias, particularly for vitamin B12 deficiency. The association between higher SII and ferritin deficiency persisted after adjustment for potential confounders. Although associations with vitamin B12 were observed in the penalized regression and continuous-outcome analyses, the univariate association did not remain statistically significant after Holm correction and the number of vitamin B12-deficient patients was limited. Accordingly, the vitamin B12 findings should be considered exploratory rather than confirmatory.

The association between systemic inflammation and iron status was most consistent for ferritin. Ferritin deficiency was associated with higher SII, NLR, and PLR values, and all three associations remained statistically significant after Holm correction for multiple comparisons (10, 25). In contrast, patients with low transferrin saturation showed higher SII and NLR values in the unadjusted analyses, but these associations did not remain statistically significant after multiple-testing correction (12). This distinction is important because ferritin reflects body iron stores but may also be influenced by inflammatory activity, whereas transferrin saturation more directly reflects circulating iron availability. Therefore, the observed findings should be interpreted as an association between systemic inflammatory indices and iron-related parameters rather than as evidence of a causal relationship (10, 24).

Accordingly, higher SII in patients with low ferritin levels may not solely reflect iron depletion itself but rather the persistent inflammatory milieu accompanying active celiac disease. This interaction supports the concept that SII captures both immune activation and nutritional disturbances instead of representing a direct marker of iron deficiency alone. Nevertheless, ferritin concentrations may also be influenced by factors unrelated to systemic inflammation, including menstrual blood loss, dietary iron intake, occult gastrointestinal bleeding, and other causes of iron deficiency. Therefore, the observed association between SII and ferritin deficiency should not be interpreted as reflecting inflammation alone but rather as the result of multiple interacting mechanisms contributing to iron homeostasis in patients with celiac disease (10, 19). Conversely, iron deficiency itself may also influence neutrophil, lymphocyte, and platelet counts, thereby contributing to changes in composite inflammation indices such as SII. Therefore, the observed association is likely to reflect the combined effects of persistent mucosal inflammation and hematological alterations related to iron deficiency rather than a single underlying mechanism.

Vitamin B12 deficiency was associated with higher SII and NLR values in the unadjusted analyses; however, these differences did not remain statistically significant after Holm correction for multiple comparisons. Exploratory analyses using Firth penalized regression and vitamin B12 as a continuous outcome nevertheless suggested a possible relationship between SII and vitamin B12 status. Although less common than iron deficiency in celiac disease, vitamin B12 deficiency may reflect more extensive intestinal involvement, prolonged malabsorption, or delayed diagnosis (25). Previous studies have primarily focused on the hematological and neurological consequences of vitamin B12 deficiency, whereas its relationship with systemic inflammation has received comparatively less attention (9). Given the limited number of vitamin B12-deficient patients and the lack of significance after multiple-testing correction in the univariate analysis, these findings should be interpreted cautiously and require confirmation in larger cohorts. The observed relationships may reflect shared underlying processes, including persistent malabsorption and mucosal immune activity, rather than a causal effect of vitamin B12 deficiency on systemic inflammation.

In contrast, no significant associations were observed between inflammation-based indices and either vitamin D or folate levels. This finding is not entirely unexpected, as the determinants of these micronutrients extend beyond intestinal absorption and inflammatory activity. Vitamin D status is influenced by multiple factors, including sunlight exposure, dietary intake, supplementation and seasonal variation, while folate levels may be affected by nutritional habits and replacement therapy (11). Consequently, the relationship between these micronutrients and systemic inflammatory markers may be less direct than that observed for iron stores. Therefore, the absence of a significant association in the present study should not be interpreted as evidence against the biological importance of vitamin D or folate in celiac disease, but rather as an indication that their circulating concentrations are determined by a broader range of factors than systemic inflammatory activity alone. Our findings suggest that not all micronutrient abnormalities in celiac disease are equally associated with systemic inflammatory indices, with the most consistent associations observed for reduced iron stores.

The present findings should be interpreted as evidence of association rather than clinical utility. Although SII was associated with ferritin deficiency in the present cohort, this study was not designed to evaluate its diagnostic performance or incremental value over conventional hematological parameters. No formal comparisons of discriminatory performance among SII, NLR, and PLR or assessments of added value beyond hemoglobin, MCV, and RDW were performed. Therefore, the present data do not establish SII as a diagnostic or adjunctive clinical marker for micronutrient deficiency. Future prospective studies specifically designed to assess diagnostic performance and incremental clinical value would be required before such a role could be considered (6, 26).

Several limitations of this study should be acknowledged. First, its retrospective and single-center design limits causal inference and may affect the generalizability of the findings. Additionally, because patients meeting the exclusion criteria were not entered into the study dataset during retrospective data collection, detailed information on the number and reasons for exclusions could not be reconstructed. Second, although disease duration and gluten-free diet adherence were available, information regarding serological activity and histopathological severity (including Marsh classification) was not consistently available and therefore could not be systematically incorporated into the analyses. These factors may influence both micronutrient status and inflammatory activity and could potentially confound the observed associations. In addition, the small number of patients with vitamin B12 deficiency limited the precision of the corresponding multivariable estimates; despite the use of penalized regression, these findings should therefore be considered exploratory. The cross-sectional nature of the study also precludes assessment of temporal relationships between changes in inflammatory indices and nutritional recovery. Furthermore, because the majority of patients were adherent to a GFD and the cohort predominantly consisted of patients under long-term follow-up, the observed inflammatory indices reflect the inflammatory status during treated follow-up rather than at the time of initial diagnosis. This limits the generalizability of our findings to newly diagnosed or untreated patients with celiac disease. Future studies with larger, prospective cohorts and more comprehensive correlation analyses across inflammatory indices and micronutrient parameters may further clarify the complex relationship between systemic inflammation and nutritional status in adult patients with celiac disease.

Despite these limitations, the study has several notable strengths. To our knowledge, this is among the few studies to comprehensively evaluate SII in relation to multiple micronutrient parameters in adult patients with celiac disease. The simultaneous assessment of ferritin, transferrin saturation, vitamin B12, folate and vitamin D levels allowed a broader characterization of nutritional status, while the inclusion of NLR and PLR provided comparator indices for contextualizing the observed associations with SII. Furthermore, all laboratory measurements were obtained from a relatively well-characterized real-world cohort using standardized methods within a single institution.

## Conclusion

In adults with celiac disease, higher systemic inflammation indices were associated with reduced iron stores, with the most consistent findings observed for ferritin deficiency. The associations of SII, NLR, and PLR with ferritin deficiency remained significant after correction for multiple comparisons, and the association between higher SII and ferritin deficiency persisted after multivariable adjustment. Although associations between SII and vitamin B12 status were observed in exploratory analyses, the univariate association did not remain significant after multiple-testing correction and should therefore be interpreted cautiously. No association was detected between systemic inflammation indices and folate or vitamin D deficiency. These findings suggest a relationship between systemic inflammatory activity and micronutrient status in treated adult celiac disease, particularly with respect to iron stores. Larger prospective studies are needed to confirm these associations and to determine their clinical relevance.

## Data Availability

The datasets generated and/or analysed during the current study are not publicly available due to institutional and patient confidentiality restrictions but are available from the corresponding author on reasonable request.
